# Structural Comparison of a Promiscuous and a Highly Specific Sucrose 6^F^-Phosphate Phosphorylase

**DOI:** 10.3390/ijms20163906

**Published:** 2019-08-11

**Authors:** Jorick Franceus, Nikolas Capra, Tom Desmet, Andy-Mark W.H. Thunnissen

**Affiliations:** 1 Centre for Synthetic Biology (CSB), Department of Biotechnology, Ghent University, Coupure Links 653, 9000 Ghent, Belgium; 2 Groningen Biomolecular Sciences and Biotechnology Institute, University of Groningen, Nijenborgh 4, 9747 AG Groningen, The Netherlands

**Keywords:** GH13_18, sucrose phosphorylase, glycoside phosphorylase, *Ilumatobacter coccineus*, *Thermoanaerobacterium thermosaccharolyticum*, crystallography

## Abstract

In family GH13 of the carbohydrate-active enzyme database, subfamily 18 contains glycoside phosphorylases that act on α-sugars and glucosides. Because their phosphorolysis reactions are effectively reversible, these enzymes are of interest for the biocatalytic synthesis of various glycosidic compounds. Sucrose 6^F^-phosphate phosphorylases (SPPs) constitute one of the known substrate specificities. Here, we report the characterization of an SPP from *Ilumatobacter coccineus* with a far stricter specificity than the previously described promiscuous SPP from *Thermoanaerobacterium thermosaccharolyticum*. Crystal structures of both SPPs were determined to provide insight into their similarities and differences. The residues responsible for binding the fructose 6-phosphate group in subsite +1 were found to differ considerably between the two enzymes. Furthermore, several variants that introduce a higher degree of substrate promiscuity in the strict SPP from *I. coccineus* were designed. These results contribute to an expanded structural knowledge of enzymes in subfamily GH13_18 and facilitate their rational engineering.

## 1. Introduction

Glycoside phosphorylases (GPs) are carbohydrate-active enzymes that catalyze the reversible cleavage of glycosidic bonds using inorganic phosphate [1,2,3]. In their physiological context, they provide a degradative route for carbohydrates and glycosides that is more energy-efficient when compared to hydrolysis. Their glycosyl phosphate reaction products can enter various pathways, such as glycolysis, without prior activation by a kinase, saving one molecule of ATP [4]. Because of the reversibility of the phosphorolytic reaction, GPs are also attractive enzymes for the synthesis of glycosidic bonds in vitro. One of the most well-studied GPs in that regard is sucrose phosphorylase (SP; EC 2.4.1.7), which catalyzes the phosphorolysis of sucrose into α-d-glucose 1-phosphate (Glc1P) and d-fructose. Thanks to the high energy content of sucrose and the enzyme’s remarkable substrate promiscuity, SP is often applied as a catalyst in cost-effective transglucosylation reactions using the cheap and renewable substrate sucrose as a glucosyl donor [5,6,7,8,9,10]. Moreover, SP has been a target in various engineering studies to further alter its specificity, selectivity, and thermostability [11,12,13,14,15].

Sucrose phosphorylase is found in subfamily 18 of glycoside hydrolase family GH13 (GH13_18) according to the Carbohydrate-Active Enzyme database (http://www.cazy.org) [16,17]. While searching this subfamily for more thermostable SPs, we previously came across a peculiar putative SP from the thermophile *Thermoanaerobacterium thermosaccharolyticum*. Although the enzyme did show significant activity on sucrose, as was expected, its kinetic parameters revealed that sucrose is in fact not its native substrate. Instead, a clear preference for sucrose 6^F^-phosphate was observed, making it the first sucrose 6^F^-phosphate phosphorylase (SPP; EC 2.4.1.329) ever reported [18]. A few key residues responsible for the difference in specificity were identified through homology modeling and mutagenesis. Furthermore, the *T. thermosaccharolyticum* SPP (TtSPP) was shown to accept an even wider range of substrates than the known SPs, offering opportunities for biotechnological applications and further engineering. For instance, a mutant of TtSPP was rationally designed to achieve high activity towards bulky phenolics such as resveratrol, enabling the quantitative production of a resveratrol glucoside [19]. The same mutant could also be applied for the synthesis of glucosylated 3-hydroxy-β-lactams [20].

The discovery of TtSPP hinted at a broader functional diversity in the family left to uncover and triggered the search for other novel GH13_18 enzymes in recent years. By characterizing proteins from unexplored branches of the phylogenetic tree, phosphorylases from *Meiothermus silvanus*, *Spirochaeta thermophila*, and *Escherichia coli* were found to be dedicated to the substrate 2-*O*-α-d-glucosylglycerate (EC 2.4.1.352) [21]. Further, a comparison of distinctive active site sequence motifs in different branches of the tree led to the discovery of a *Marinobacter adhaerens* enzyme that acts as a configuration-retaining 2-*O*-α-d-glucosylglycerol phosphorylase (EC 2.4.1.359) [22]. In this work, we report the expression and characterization of an SPP from *Ilumatobacter coccineus* YM16–304 (IcSPP) with active site sequence motifs that are different from the motifs found in TtSPP or in the phosphorylases with other specificities in the GH13_18 subfamily. The enzyme shows high activity on sucrose 6^F^-phosphate, but unlike TtSPP, it does not catalyze reactions with alternative substrates. Crystal structures of the two SPPs were determined to allow for a comparison of their active site. Automated docking and mutational analyses were carried out to obtain variants of IcSPP with a less stringent specificity.

## 2. Results and Discussion

### 2.1. Choice of the Target Sequence

**Figure 1:**
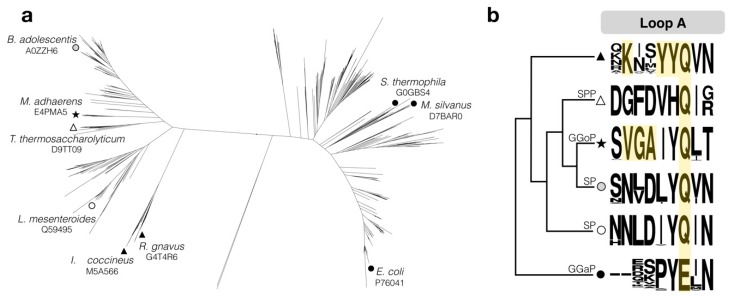
(**a**) Phylogenetic tree of protein sequences classified in subfamily GH13_18. A selection of representatives that have been characterized and reported in the literature are indicated with their UniProt ID. (**b**) Simplified phylogenetic tree showing the sequence logo of an acceptor site loop for the proteins in the branch. Motifs mentioned in the main text are highlighted. Black triangle, IcSPP-like sucrose 6^F^-phosphate phosphorylases (target clade in this study); white triangle, TtSPP-like sucrose 6^F^-phosphate phosphorylases; star, glucosylglycerol phosphorylases (GGoP); grey circle, *Bifidobacterium*-like sucrose phosphorylases; white circle, lactic acid bacteria-like sucrose phosphorylases; black circle, glucosylglycerate phosphorylases (GGaP).

As knowledge on the diversity of substrate specificities in subfamily GH13_18 expanded, a few sequence motifs that are highly conserved among enzymes sharing the same specificity could be identified. Such signature motifs were first detected in sucrose 6^F^-phosphate phosphorylases and glucosylglycerate phosphorylases and were then utilized to predict that the putative sucrose phosphorylase from *M. adhaerens* is actually a glucosylglycerol phosphorylase [18,21,22]. Perhaps the most distinct specificity-determining region is the so-called loop A, found in domain B’ between strand β7 and helix α7 of the catalytic domain (Figure 1). For example, one position in that loop carries a glutamine residue in all enzymes except glucosylglycerate phosphorylases, where glutamate is present instead. Likewise, glucosylglycerol phosphorylases possess a conserved VGA motif that is absent in all other enzymes.

Another clade of interesting enzymes was encountered by thoroughly searching the subfamily’s phylogenetic tree for more sequences with an aberrant motif in loop A. These atypical sequences feature several notable conservations that are not found in any other clades, such as a YYQ motif and a lysine at a position where others hold glycine, valine, or asparagine. The enzymes in the target clade primarily originate from the microbial classes *Clostridia*, *Flavobacteriia*, and *Acidimicrobiia*. We set out to elucidate their properties and selected a candidate from *Ilumatobacter coccineus* YM16–304 for expression and characterization. Very recently, Tauzin et al. already reported the characterization of an enzyme from the gut bacterium *Ruminococcus gnavus* that shows the same characteristic residues in loop A [23]. That enzyme, which has 52% sequence identity to the homologue from *Ilumatobacter coccineus* presented here, was found to be an SPP with a very strict specificity.

### 2.2. Expression and Characterization of IcSPP

IcSPP, provided with a C-terminal His_6_-tag, was recombinantly expressed in *E. coli* and purified to apparent homogeneity by affinity chromatography. Approximately 12 mg of purified protein was obtained from a 250 mL culture. The protein migrated in sodium dodecyl sulfate-polyacrylamide gel electrophoresis as a single band with an apparent molecular mass that is in accordance with the theoretical mass deduced from the amino acid sequence (59.9 kDa; Figure S1).

**Figure 2:**
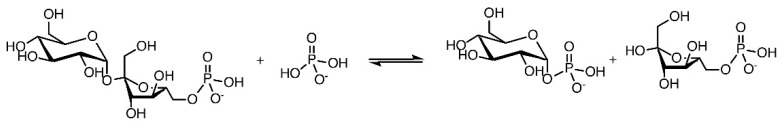
Reaction catalyzed by sucrose 6^F^-phosphate phosphorylase.

The activity in synthetic direction was determined for more than 30 different putative acceptor substrates, using Glc1P as glucosyl donor (Table S1). When fructose 6-phosphate was added, the measured specific activity (45 ± 3 U/mg) was in the same range as reported wild-type activities of other GH13_18 phosphorylases. However, no activity was observed on any of the other compounds. These results indicate that the enzyme is a sucrose 6^F^-phosphate phosphorylase with strict specificity (Figure 2), consistent with the earlier characterization of the homologous *R. gnavus* SPP by Tauzin et al. [23]. Reactions in the physiologically relevant degradative direction confirmed the high activity on sucrose 6^F^-phosphate (53 ± 4 U/mg) and the lack of activity on other compounds. IcSPP and the *R. gnavus* SPP described previously thus behave very differently from the promiscuous *T. thermosaccharolyticum* SPP (TtSPP) that resides in a different clade of the subfamily’s phylogenetic tree.

The biochemical properties of IcSPP were investigated. The optimal pH in the synthesis direction of the reactions was 6.5, and more than 50% of the maximum activity was retained within the pH range of 5.5 to 8 (Figure S2A). In the phosphorolytic direction, the optimum was reached at pH 6. Optimal activity was achieved at a temperature of 35 °C (Figure S2B), which is in line with the mesophilic nature of the *Ilumatobacter* strain that the protein originates from [24]. The enzyme followed Michaelis–Menten kinetics at the tested substrate concentrations (Table 1). Notably, the affinity for fructose 6-phosphate appears to be considerably higher in IcSPP (*K*_M_ 2.0 ± 0.3 mM) than in TtSPP (*K*_M_ 15.1 ± 2.3 mM).

### 2.3. Structural Comparison of IcSPP and TtSPP

**Figure 3:**
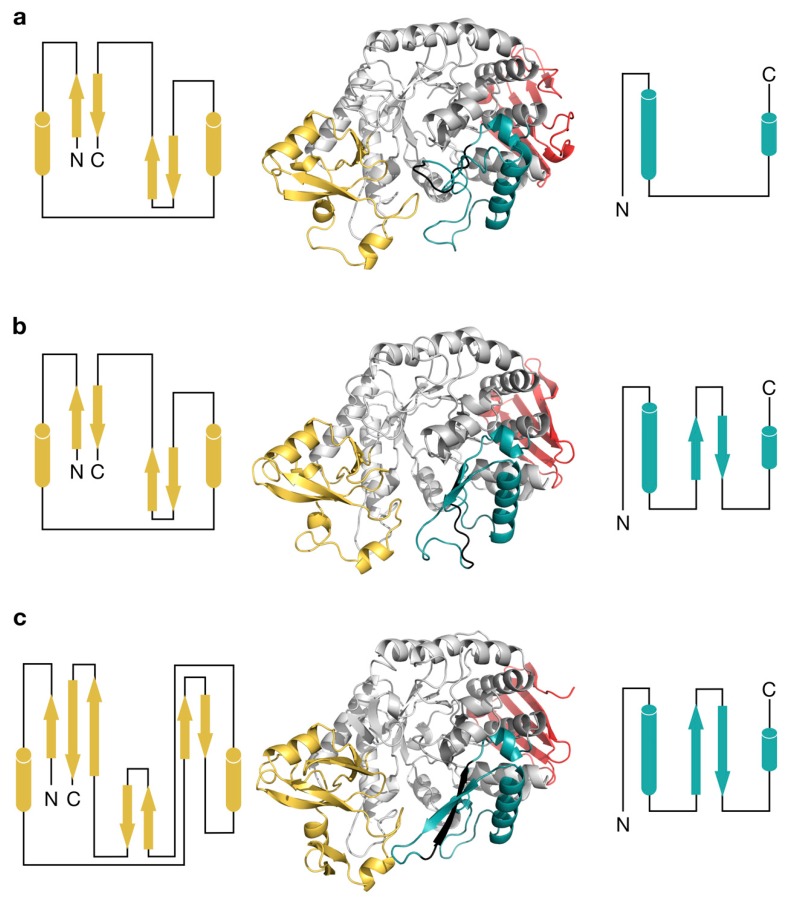
Differences in domains B (yellow) and B’ (teal) of (**a**) BaSP, (**b**) TtSPP, and (**c**) IcSPP. The C-terminal domain is shown in red, loop A within domain B’ is shown in black. Schematic secondary structure topology diagrams for loop B and B’ are shown left and right of the structure, respectively.

The crystal structures of the SPPs from *I. coccineus* and *T. thermosaccharolyticum* were determined to 2.05 Å and 1.83 Å resolution, respectively (Table S2; PDB codes 6S9U and 6S9V). The IcSPP crystals contained one protein molecule in the asymmetric unit, whereas the TtSPP crystals contained two. Overall, the monomers showed high structural similarity to the *Bifidobacterium adolescentis* sucrose phosphorylase (BaSP; PDB codes 2GDU, 2GDV, 1R7A), the only other GH13_18 enzyme of which a three-dimensional structure is currently known [13,25,26], despite the limited sequence identity of TtSPP and IcSPP to BaSP (35.1% and 26.3%, respectively). Four distinct domains can be discerned in these three enzymes. Domain A (residues 1–93, 198–328, and 389–470 in IcSPP) is the largest and forms the (β/α)_8_-barrel that is characteristic to the GH13 family. The domain at the C-terminal end (residues 471–523) is made up of a five-stranded antiparallel β-sheet. However, the backbone conformation of the two remaining domains B (residues 94–197) and B’ (residues 329–388) appears to be more variable (Figure 3). Domain B in BaSP and TtSPP contains two antiparallel β-sheets formed by two strands each, flanked by two short α-helices. In IcSPP, the inner sheet immediately flanking the barrel is considerably larger and formed by three strands instead of two. IcSPP also has an additional set of two strands constituting a third, outermost β-sheet. Domain B’ in BaSP is a coil region with two α-helices. In both SPPs, however, part of the coil region is replaced by a β-sheet made out of antiparallel strands. Furthermore, those strands are larger in IcSPP than they are in TtSPP.

IcSPP seems to exist as a monomer, whereas TtSPP in the crystal structure forms dimers. BaSP was also shown to form dimers, but the arrangements of the TtSPP and BaSP dimers are quite different (Figure S3), and the TtSPP dimer interface is somewhat smaller compared to that of BaSP (780 Å^2^ versus 960 Å^2^). Based on size exclusion chromatography and light scattering analysis, both IcSPP and TtSPP, unlike BaSPP, are present as monomers in solution at pH 7. Thus, the dimers observed in the TtSPP crystals probably do not represent a functional state of the protein.

**Figure 4:**
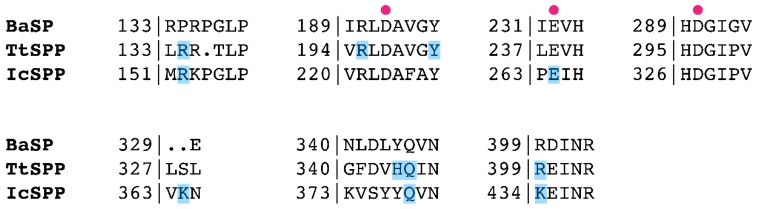
Partial multiple sequence alignment of BaSP, TtSPP, and IcSPP. Catalytic residues are indicated by a purple sphere. Residues suggested to be involved in binding of fructose 6-phosphate, according to automated docking, are highlighted in blue.

**Figure 5:**
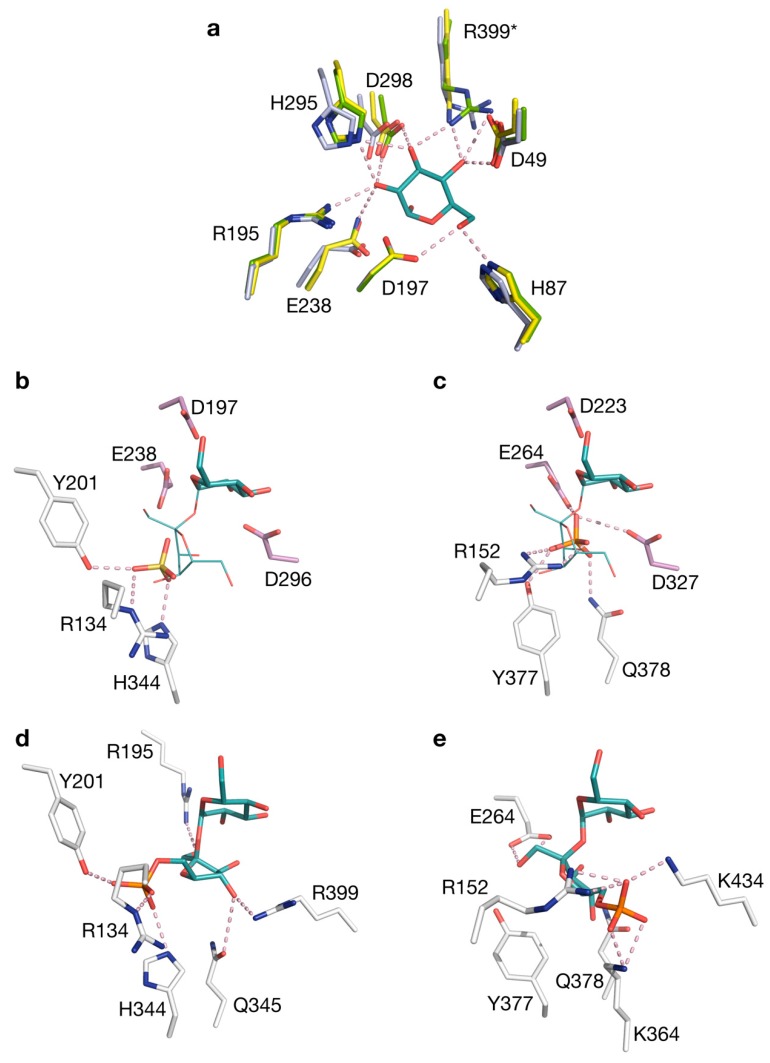
Active site of sucrose 6^F^-phosphate phosphorylases (SPPs). (**a**) Overlay of the −1 subsite of BaSP (green), TtSPP (blue), and IcSPP (yellow), with the glucosyl moiety of sucrose from the crystal structure of BaSP (PDB code 2GDU). Residue numbering according to TtSPP. Arg399 is substituted by Lys in IcSPP (asterisk). (**b**) Close-up view of the active site in TtSPP with a bound sulfate ion. (**c**) Close-up view of the active site in IcSPP with a bound phosphate ion. The catalytic residues are shown in purple. An overlay of sucrose from the structure of BaSP is shown for reference. Automated docking of sucrose 6^F^-phosphate in (**d**) TtSPP and (**e**) IcSPP. All figures of TtSPP were made using molecule B from the asymmetric unit of the crystal structure.

A closer look was taken at the sequence and structure of the active site (Figure 4, Figure 5, Figure S4). It seems that the architecture of subsite −1 is essentially identical in BaSP and the SPPs and most likely across all GH13_18 enzymes. Subsite −1 is dedicated to the recognition of the glucosyl moiety of the donor substrate. Residues that form hydrogen bonds to the glucosyl group are highly conserved, both in sequence and in structure (Asp49, His87, Arg195, His295, Arg399 in TtSPP) (Figure 5a). The same is evidently true for the catalytic nucleophile and general acid/base at the tips of β-sheets 4 and 5 of the (β/α)_8_-barrel, and the aspartate that crucially stabilizes the transition state. These catalytic residues are, respectively, found at Asp197, Glu238, and Asp296 in TtSPP. In IcSPP, they are found at Asp223, Glu264, and Asp327. Only the arginine that interacts with OH3 and OH4 is an exception to the strict conservation in subsite −1 as it is substituted by lysine in IcSPP.

One tetrahedral sulfate ion is present in subsite +1 of TtSPP. It is held by hydrogen bonds with Tyr201, Arg134, and His344 (Figure 5b). Mutagenesis previously confirmed that the latter two are involved in binding the phosphate moiety of sucrose 6^F^-phosphate [18]; hence, the sulfate group in the crystal structure is probably situated at that particular binding site. In the structure of IcSPP, a phosphate ion is found interacting with Arg152, Glu264, Asp327, Tyr377, and Gln378 in the active site. One of the oxygen atoms of phosphate is in close proximity to the likely position of the anomeric carbon of the glucosyl donor. This observation suggests that the complexed molecule takes on the binding mode of the phosphate that attacks the covalent glucosyl-enzyme intermediate during the enzyme’s double displacement reaction (Figure 5c) [27,28].

Automated docking was performed to predict the binding mode of sucrose 6^F^-phosphate in the SPP structures. For both enzymes, a cluster could be obtained where the glucosyl moiety of the substrate was located in subsite −1, in full agreement with the structure of BaSP in complex with sucrose. The anomeric carbon atom, as well as the oxygen atom of the glycosidic linkage, were at an appropriate distance and angle from the catalytic residues. Although the possibility of conformational rearrangements upon binding must be considered, several residues that participate in hydrogen bonding with the fructose 6-phosphate moiety could be identified in subsite +1, and these residues are fully conserved among sequences from the same phylogenetic branch. Arg134, Arg195, Tyr201, His344, Gln345, and Arg399 fulfill this role in TtSPP (Figure 5d). Furthermore, the position of the substrate’s phosphate group indeed matches the position of the sulfate molecule that was seen in the crystal structure. In IcSPP, the predicted binding partners are Arg152, Glu264, Lys364, Gln378, and Lys434 (Figure 5e). Clearly, the set of amino acids responsible for substrate binding in subsite +1 is different between the two enzymes. For instance, Lys364 of IcSPP is considered to be properly situated to interact with fructose 6-phosphate, while no obvious function is predicted for the corresponding serine residue in TtSPP. As another example, IcSPP houses a tyrosine at position 377, just like sucrose phosphorylases do at the equivalent position. In TtSPP, on the other hand, a histidine residue is present instead. Mutating this histidine into tyrosine lowers the activity of TtSPP on fructose 6-phosphate almost fivefold [18]. Finally, it is worth noting that one of the strictly conserved residues in loop A of IcSPP-like enzymes, Lys373 (Figure 1b), is not among the predicted substrate-binding residues. Its sidechain is pointed away from the active site in the crystal structure (Figure S5), just like the conserved Asn residue that is present at the corresponding position in SP. The reason for their conservation remains unknown for now, although it is conceivable that they play a role in the backbone rearrangements that loop A undergoes during catalysis [26].

### 2.4. Mutagenesis

Despite the structural differences, an obvious reason for the strict specificity of IcSPP in contrast to the promiscuous behavior of TtSPP is not readily apparent from their active site layout alone. Therefore, the possibility of introducing alternative activities in IcSPP by means of mutagenesis was explored. We hypothesized that by disrupting the interaction network with the phosphate group from sucrose 6^F^-phosphate, the acceptor site might recognize compounds lacking that phosphate group and take on a more relaxed shape in general. The residues participating in hydrogen bonds with the phosphate, according to the docking analysis, are Arg152, Lys364, and Lys434. Tyr377 was also targeted because of its close proximity, although its role in substrate binding is more ambiguous. All residues were substituted by the amino acid that is found at the equivalent position in TtSPP. However, position 152 is occupied by arginine in both SPPs and was therefore mutated into alanine to remove the side chain while retaining the structural integrity of the backbone [29]. An additional alanine mutant was also created at position 434 due to the similar properties of the lysine and arginine sidechains found in IcSPP and TtSPP, respectively.

All mutants were evaluated in the synthesis direction, using α-d-glucose 1-phosphate as the glucosyl donor (Table 2). Determination of the kinetic parameters for glucosyl acceptor fructose 6-phosphate pointed out that all mutants suffer a pronounced drop in activity and/or affinity. Mutation K434R seemed to be the least detrimental in terms of activity, as was expected. However, the most drastic effect on turnover rate and affinity was observed when the same residue was mutated into alanine. The affinity of mutant Y377H for fructose 6-phosphate decreased only slightly, supporting the results of the docking experiment.

To assess the ability of the mutants to employ alternative substrates, their activity was measured using fructose, glycerol, and d-glycerate as the glucosyl acceptor (Table 2). Those reactions represent the other known wild-type activities that have been discovered in subfamily GH13_18 thus far. Only variant Y377H, mutated at a position that was not predicted to interact with the phosphate group of fructose 6-phosphate, was unable to perform any of the alternate transglucosylation reactions. All others could effectively use fructose to synthesize sucrose. Mutants R152A and K364S also exhibited activity on glycerol and d-glycerate. However, the substitutions at position 434 had a dissimilar effect in that regard. Mutant K434R could use the negatively charged d-glycerate but not glycerol, whereas the opposite was true for K434A. Considering the properties of these variants, it can be concluded that the breadth of acceptor substrates tolerated by IcSPP can be extended rather easily. Although the measured activities may sound modest, being merely up to a few times higher than the hydrolytic side-activity that is inherent to the enzyme’s double displacement mechanism, they are not insignificant. For reference, the ratio of the transglucosylation activity with glycerol over the hydrolytic activity ranges between 1.2 and 1.7 with *Leuconostoc mesenteroides* SP [6], yet this transglucosylation process was eventually optimized for the production of glucosylglycerol on a commercial scale [30,31].

## 3. Conclusions

The structures of the highly specific sucrose 6^F^-phosphate phosphorylase from *Ilumatobacter coccineus* and its exceptionally promiscuous homologue from *Thermoanaerobacterium thermosaccharolyticum* provided insight into their similarities and differences. The architecture of subsite +1 showed the most prominent disparities, where a different set of residues appears to be responsible for substrate recognition. Mutational analysis revealed that the specificity of the *I. coccineus* phosphorylase can be loosened up by targeting positions around its predicted phosphate group binding site. More specifically, single point substitutions at positions Arg152, Lys364, or Lys434 were sufficient to introduce activity on fructose, glycerol, or d-glycerate, the natural acceptor substrates of related GH13_18 enzymes. This study finally expanded our structural knowledge of GH13_18, as the three-dimensional structure of only one enzyme from this subfamily has been described so far [25]. The findings also open up perspectives for further engineering of SPPs and other GH13_18 phosphorylases. Indeed, it has been established that engineering efforts can be more successful when starting from a collection of different scaffolds [32,33]. TtSPP was already proven to be a favorable starting point for mutagenesis [19], and the availability of a crystal structure should promote further exploration of its capabilities. Moreover, considering how the strict behavior of IcSPP can be loosened up, the same might be true for the related glucosylglycerol and glucosylglycerate phosphorylases. These relaxed enzymes may then turn out to be appealing templates for designing catalysts for the synthesis of various valuable glucosides and sugars as well. 

## 4. Materials and Methods 

### 4.1. Materials

All chemicals were obtained from Sigma-Aldrich, Merck or Carbosynth unless noted otherwise and were of the highest purity. α-D-Glucose 1-phosphate was produced in house using sucrose phosphorylase [34]. The acid glucose 1-phosphatase negative strain *Escherichia coli* CGSC 8974 was obtained from the Coli Genetic Stock Center. The *E. coli* BL21(DE3) strain was obtained from New England Biolabs.

### 4.2. Sequence Analysis

All 2405 full-length protein sequences in family GH13, subfamily 18, were extracted from the CAZy database (http://www.cazy.org) [17] and subsequently aligned with ClustalO using default settings [35]. A script was written in Python to remove duplicate sequences, retaining only 1254 unique sequences. A maximum-likelihood phylogenetic tree was constructed using PhyML 3.1 with default parameters [36] and visualized using iTOL v4 [37]. Sequence logos were generated with WebLogo [38]. Multiple sequence alignments were visualized using ESPript 3.0 [39].

### 4.3. Gene Cloning and Transformation

The *T. thermosaccharolyticum* sucrose 6^F^-phosphate phosphorylase (UniProt code D9TT09) was expressed as described in earlier work [18]. The amino acid sequence for the *I. coccineus* phosphorylase (UniProt code M5A566) was codon-optimized for *E. coli,* synthesized, and subcloned into a pET21a vector by restriction digestion with *Nhe*I and *Xho*I and ligation by Life Technologies (Merelbeke, Belgium). The plasmid was transformed in *E. coli* CGSC 8974 electrocompetent cells. Sequences are listed in Table S3.

### 4.4. Protein Expression and Purification

TtSPP was expressed and purified as described in earlier work [18]. To express IcSPP, 2% of an overnight culture was inoculated in 500 mL LB medium containing 100 µg/mL ampicillin in a 2 L Erlenmeyer flask and grown at 37 °C with continuous shaking at 200 rpm. The culture was incubated until OD_600_ reached ~0.6. Then the temperature was lowered to 18 °C, and expression was induced by adding 0.1 mM isopropyl β-D-1-thio-galactopyranoside. Protein expression took place for 18 h. The culture was spun down, and the pellet was frozen at −20 °C for at least 4 h.

Cell pellets of a 250 mL culture were thawed at 4 °C and dissolved in 8 mL lysis buffer that consists of 0.1 mM phenylmethylsulfonyl fluoride, 1 mg/mL lysozyme, 10 mM imidazole, and 50 mM phosphate buffered saline. After incubation on ice for 30 min, the lysate was sonicated 3 times for 3 min (Branson Sonifier 250, level 3, 50% duty cycle) and the resulting extract was centrifuged (9000 rpm, 1 h, 4 °C). The supernatant was further purified by means of nickel-nitrilotriacetic acid chromatography as described by the supplier (Thermo Scientific, Waltham, MA, USA), and the buffer was exchanged to 2-morpholinoethanesulfonic acid (MES) buffer (pH 6.5) in a 30 kDa Amicon Ultra centrifugal filter (Merck Millipore, Darmstadt, Germany). A NanoDrop ND-1000 (Thermo Scientific, Waltham, MA, USA) was applied to measure the protein concentration in triplicate, using the extinction coefficients calculated with the ProtParam tool on the ExPASy server (http://web.expasy.org/protparam/). Molecular weight and purity were verified by sodium dodecyl sulfate-polyacrylamide gel electrophoresis (10% gel).

### 4.5. Site-Directed Mutagenesis

Site-directed mutations were introduced with a megaprimer-based whole-plasmid PCR method described elsewhere [40]. The forward primer contained the desired mutation, whereas the reverse primer (pET21a_Rv_seq1) was kept constant (Table 3). Template DNA was digested by *Dpn*I treatment (Westburg, Leusden, Netherlands) for at least 3 h at 37 °C. After transformation in *E. coli* BL21(DE3) electrocompetent cells, the obtained plasmid was subjected to nucleotide sequencing to verify the presence of the mutation (Macrogen, Amsterdam, Netherlands).

### 4.6. Colorimetric Assays

The activity of IcSPP and TtSPP was measured in the phosphorolysis direction of the reversible reaction by measuring the reduction of NAD^+^ in the presence of phosphoglucomutase and glucose 6-phosphate dehydrogenase (glucose 1-phosphate assay) [41]. In the synthesis direction, the release of inorganic phosphate from glucose 1-phosphate could be quantified with the phosphomolybdate assay [42]. To determine the hydrolytic side-activity, an enzymatic coupled assay with glucose oxidase and peroxidase was carried out (GOD-POD assay) [43]. The true transglucosylation activity could be calculated by subtracting the hydrolytic activity from the total activity in synthesis direction, as determined by the phosphomolybdate assay. Samples were inactivated by the acidic conditions of the assay solution (phosphomolybdate assay) or by heating for 5 min at 95 °C (other assays).

### 4.7. Characterization of IcSPP

To assess which acceptor substrates IcSPP is active on, ~50 µg/mL enzyme was incubated with 50 mM α-d-glucose 1-phosphate (Glc1P) and 100 mM acceptor in 50 mM MES buffer (pH 6.5) for 30 min at 30 °C in a total reaction volume of 1 mL. A sample was taken and analyzed with the phosphomolybdate and GOD-POD assays. To assess the donor substrates, ~50 µg/mL enzyme was incubated with 50 mM donor and 50 mM phosphate under the same conditions, and a sample was analyzed with the Glc1P assay. To precisely quantify the specific activity of IcSPP towards its wild-type substrates, 5 µg/mL enzyme was added. The reactions were carried out at 37 °C for 8 min (reaction volume of 1 mL), and a sample was taken and analyzed every min.

The influence of pH on activity was checked in the synthesis direction in 50 mM acetate (pH 4.5), MES (pH 5.0–6.5), or 3-(N-morpholino)propanesulfonic acid (pH 7.0–8.0) at 37 °C, and the optimal temperature was determined in 50 mM MES, pH 6.For each reaction, 5 μg/mL enzyme was incubated with 50 mM Glc1P and 50 mM fructose 6-phosphate (reaction volume of 1 mL). Samples of 50 μL were taken every 30 s for 4 min and analyzed with the phosphomolybdate and GOD-POD assays.

The apparent kinetic parameters of IcSPP for its natural substrates were determined at the optimal temperature and pH in 50 mM MES buffer (reaction volume of 1 mL), with samples taken every minute for 8 min. The enzyme concentration was 4 μg/mL, and the fixed cosubstrate was either 100 mM sucrose 6^F^-phosphate or 100 mM phosphate in the degradation direction, or either 100 mM fructose 1-phosphate or 100 mM Glc1P in the synthesis direction. When evaluating the IcSPP mutants, the enzyme concentration was increased to 30 µg/mL (K434R) or 400 µg/mL (others) due to their lower activity. Parameters were calculated by non-linear regression of the Michaelis–Menten equation using SigmaPlot 11. The molecular weight of 55.9 kDa was used to calculate the turnover number *k*_cat_.

The activity of IcSPP mutants on the alternative acceptor substrates was measured at 30 °C and in 50 mM MES buffer (pH 6.5), with 500 µg/mL enzyme, 50 mM Glc1P, and either 30 mM fructose 6-phosphate, 200 mM fructose, 200 mM glycerol, or 50 mM d-glycerate (reaction volume of 1 mL). Samples were taken every 2 min for 16 min and analyzed with the phosphomolybdate assay.

### 4.8. Crystallography

Crystallization screening was preceded by an extra protein purification step using a Superdex 200 10/300 GL column (GE Healthcare, Buckinghamshire, UK) preequilibrated with 100 mM Tris buffer (pH 7.5) for IcSPP or 20 mM Bis-Tris (pH 7.5) and 150 mM NaCl for TtSPP. Both proteins eluted as monomers with an apparent molecular weight of about 60 kDa, as confirmed by dynamic light scattering analysis. Purified IcSPP and TtSPP were concentrated to 9 mg/mL in 10 mM Tris (pH 7.5) and 6 mg/mL in 10 mM Bis-Tris (pH 7.5) with 75 mM NaCl, respectively. Screening for crystallization conditions was performed at room temperature in 96-well sitting-drop crystallization plates, using various commercial crystallization screens and the help of a Mosquito (TTP LabTech) pipetting robot. Drops were prepared by mixing 200 nL protein solution with 200 nL reservoir solution. Crystals for IcSPP were obtained in the presence of 20 mM fructose 6-phosphate with a reservoir solution containing 20% PEG 3350, 200 mM Na/K phosphate, and 100 mM Bis-Tris propane buffer (pH 7.5), while crystals for TtSPP grew in the presence of 40 mM fructose 6^F^-phosphate with a reservoir solution containing 26% PEG 3350, 100 mM (NH_3_)_2_SO_4_, and 100 mM Bis-Tris propane buffer (pH 5.5). Although fructose 6^F^-phosphate was present in the crystallization trials, no sugar could be detected in the final structures. Diffraction data for IcSPP and TtSPP were recorded at the synchrotron (ESRF, Grenoble). Prior to X-ray data collection, the crystals were flash-cooled in liquid nitrogen using 20% PEG400 as a cryo-protectant. Processing of the X-ray diffraction data was performed with the program XDS [44] and with the AIMLESS routines of the CCP4 software suite [45]. The IcSPP crystals belong to space group C222_1_ and contain a single polypeptide chain in the asymmetric unit (solvent content of 44%), whereas the TtSPP crystals belong to space group P2_1_2_1_2_1_ and contain two polypeptide chains in the asymmetric unit (solvent content of 42%). Initial phases and electron maps were obtained by molecular replacement with the program Phaser [46], using the structure of BaSP (PDB code 1R7A) as a search model. The resulting models were improved by automatic model building using ARP/wARP [47], followed by several rounds of model building and refinement, using the program Coot [48] and routines from the Phenix software suite [49]. Validation of the final structures was performed with the Molprobity server [50]. 

### 4.9. Automated Docking and Protein Figures

The binding of sucrose 6^F^-phosphate in IcSPP and TtSPP was simulated by ligand docking using the AutoDock VINA module implemented in YASARA [51]. The default settings were applied, except for the number of runs which was increased to The correct cluster was selected by comparing the binding mode of the ligand’s glucosyl moiety in subsite −1 to the binding mode observed in BaSP (PDB code 2GDU).

All structure manipulations, such as superpositions, were carried out in YASARA. Figures were rendered in PyMOL [52].

## Figures and Tables

**Table 1:** Apparent kinetic parameters of *I. coccineus* sucrose 6^F^-phosphate phosphorylase at 35 °C and 50 mM 3-(N-morpholino)propanesulfonic acid (MOPS) pH 7.0 (phosphorolysis) or 50 mM MES pH 6.5 (synthesis).

Reaction	Substrate	*K*_M_ (mM)	*k*_cat_ (s^−1^)	*k*_cat_/*K*_M_ (mM^−1^s^−1^)
Phosphorolysis	sucrose 6^F^-phosphate	11.3 ± 1.8	126 ± 15	11.2
	phosphate	7.8 ± 1.2	110 ± 9	14.1
Synthesis	α-d-glucose 1-phosphate	18.8 ± 3.6	45 ± 3	2.4
	fructose 6-phosphate	2.0 ± 0.3	40 ± 4	20

**Table 2:** (Left) Apparent kinetic parameters of *I. coccineus* sucrose 6^F^-phosphate phosphorylase mutants using 50 mM glucose 1-phosphate at 35 °C and pH 6.5. (Right) Transglucosylation activity using 50 mM glucose 1-phosphate and 30 mM (fructose 6-phosphate; Fru6P), 50 mM (d-glycerate), or 200 mM (others) acceptor at 30 °C and pH 6.5, expressed with hydrolysis as reference.

Mutant	Kinetics Fru6P		*v*_acceptor_ */v*_water_			
	*K*_M_ (mM)	*k*_cat_ (s^−1^)	Fru6P	Fructose	Glycerol	d-Glycerate
Wild-type	2.0 ± 0.3	40.0 ± 3.8	1360 ± 120	-	-	-
R152A	3.3 ± 0.7	1.42 ± 0.11	98 ± 5	6.9 ± 0.3	2.4 ± 0.3	2.5 ± 0.2
K364S	6.3 ± 1.1	0.61 ± 0.04	18 ± 2	1.3 ± 0.1	1.3 ± 0.1	1.3 ± 0.1
Y377H	2.8 ± 0.3	0.80 ± 0.08	21 ± 2	-	-	-
K434R	10.6 ± 1.9	6.84 ± 0.15	680 ± 45	8.1 ± 0.4	-	4.9 ± 0.4
K434A	33.2 ± 2.9	0.10 ± 0.01	3.5 ± 0.5	2.8 ± 0.4	1.4 ± 0.2	-

(-): No significant transglucosylation activity could be detected.

**Table 3:** Primers used in this study. Mutations are underlined.

Primer	DNA sequence (5′-3′)
IcSPP_Fw_R152A	CGTCTGTTTATGGCTAAACCGGGTCTGC
IcSPP_Fw_K364S	GGTGGTCGTGTGAGTAATCTGTATGGTG
IcSPP_Fw_Y377H	GGTACAAAAGTGAGCTATCATCAGGTTAACGCC
IcSPP_Fw_K434R	GGTGCGGATGGTCATCGTGAAATCAATCG
IcSPP_Fw_K434A	GGTGCGGATGGTCATGCAGAAATCAATCG
pET21a_Rv_seq1	TCCGCGCACATTTCC

## Supplementary Materials

Supplementary materials can be found at https://www.mdpi.com/1422-0067/20/16/3906/s1.

## Abbreviations

BaSP	*Bifidobacterium adolescentis* sucrose phosphorylase
Fru6P	Fructose 6-phosphate
GGaP	Glucosylglycerate phosphorylase
GGoP	Glucosylglycerol phosphorylase
GH13_18	Subfamily 18 of glycoside hydrolase family 13
Glc1P	α-d-glucose 1-phosphate
GOD-POD	Glucose oxidase—peroxidase
GP	Glycoside phosphorylase
IcSPP	*Ilumatobacter coccineus* sucrose 6^F^-phosphate phosphorylase
MES	2-morpholinoethanesulfonic acid
MOPS	3-(N-morpholino)propanesulfonic acid
SP	Sucrose phosphorylase
SPP	Sucrose 6^F^-phosphate phosphorylase
TtSPP	*Thermoanaerobacterium thermosaccharolyticum* sucrose 6^F^-phosphate phosphorylase
