# Supplementary material for: Structural Comparison of a Promiscuous and a Highly Specific Sucrose 6F-Phosphate Phosphorylase
Source: Int J Mol Sci. 2019 Aug 11;20(16):3906. doi: 10.3390/ijms20163906 (PMC6720575; doi:10.3390/ijms20163906)
Supplement: Supplementary file 1 [file ijms-20-03906-s001.pdf]

Supplementary Information for

Structural comparison of a promiscuous and a highly specific sucrose 6^F-phosphate phosphorylase

International Journal of Molecular Sciences

Jorick Franceus, Nikolas Capra, Tom Desmet, and Andy-Mark W.H. Thunnissen

Corresponding author:
Jorick Franceus, Centre for Synthetic Biology
Email: jorick.franceus@ugent.be
Website: www.biocatalysis.ugent.be

CONTENTS

Fig. S1 SDS-PAGE . 2

Fig. S2 Effect of pH and temperature on IcSPP activity. 2

Fig. S3 Superposition of homodimeric structures. 3

Fig. S4 Multiple sequence alignment. 4

Fig. S5 Position of Lys373 in the structure of IcSPP. 5

Table S1 Compounds evaluated as substrates of IcSPP. 6

Table S2 Data collection and refinement statistics 7

Table S3 Protein sequences of IcSPP and TtSPP. 8

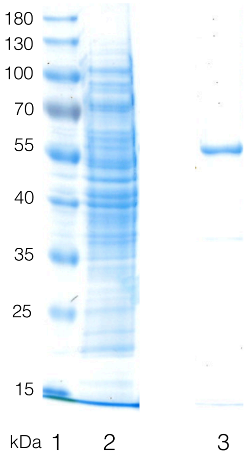

Figure S1 SDS-PAGE for samples of IcSPP. (1) PageRuler prestained protein ladder (ThermoFisher), (2) cell-free extract, (3) purified fraction.

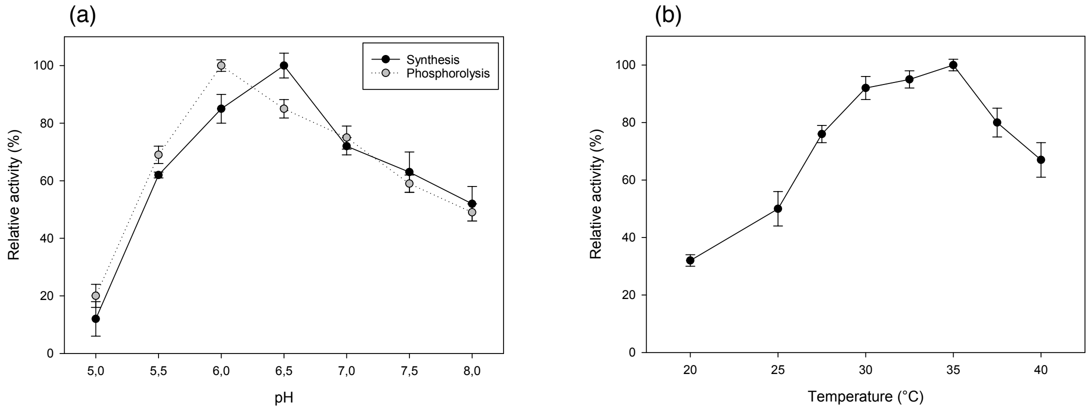

Figure S2 The effect of (a) pH and (b) temperature on IcSPP activity. The pH profile was determined in the synthesis (black circles) and the phosphorolytic (grey circles) directions; the temperature profile was determined in the synthesis direction.

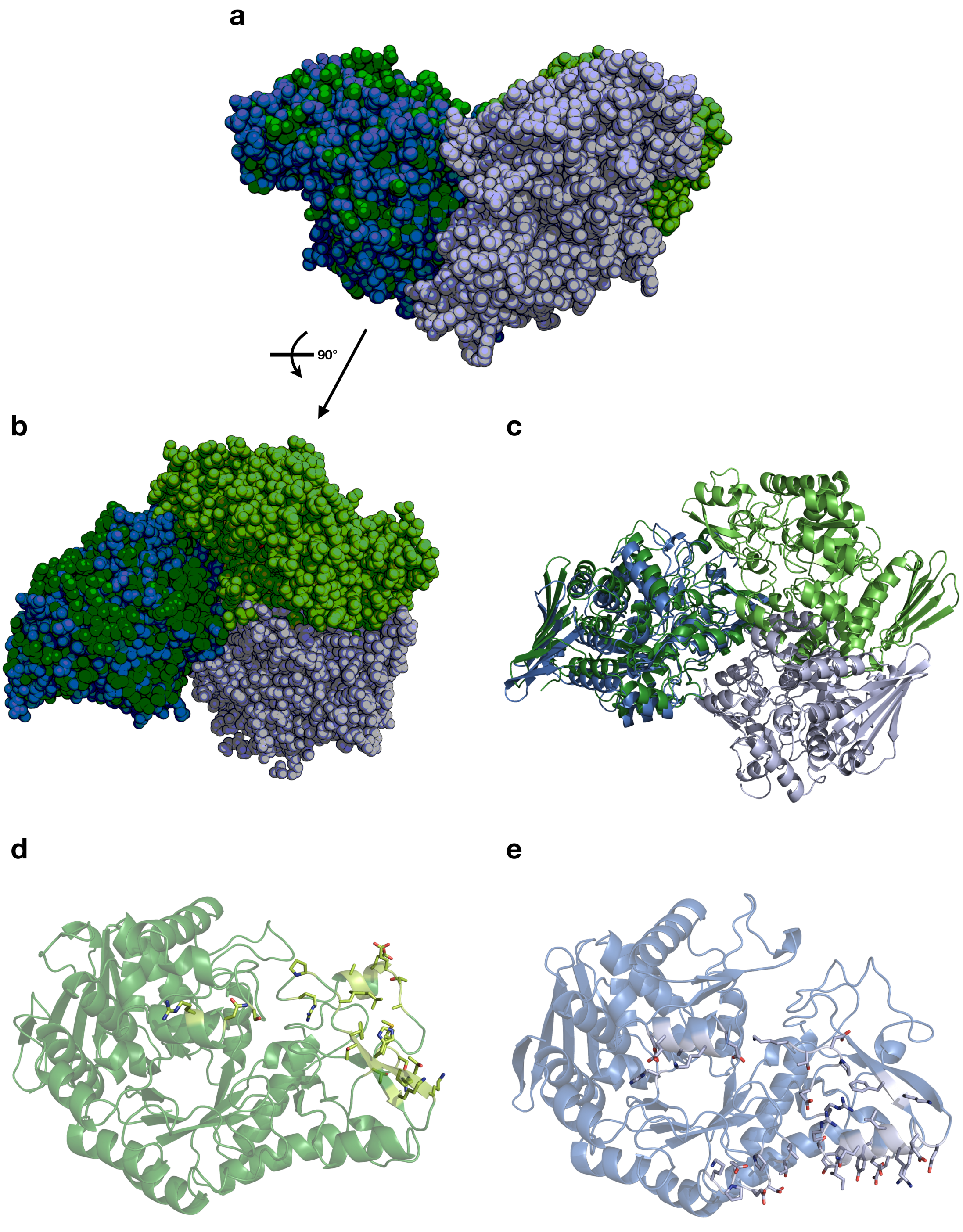

Figure S3 The BaSP (green) and TtSPP (blue) homodimeric structures with molecule B superposed, shown as spheres (a, b) or in cartoon representation (c). One monomer of BaSP (d) and TtSPP (e) with the residues at the dimer interface shown as sticks.

```
BaSP      1   .........MKNKVQLITYADRLGDGTIKSMTDILRTRFDGVYDGVHILPFFTPFDGADA
TtSPP     1   .......MALKNKVQLITYPDSLG.GNLKTLNDVLEKYFSDVFGGVHILPPFPS..SGDR
IcSPP     1   MTHAGMKASLPNRVMLNAYPDSIDGDLAGTVRMLQRPEFTDAFGLFYVLPSIFN.SDLDR

BaSP     52   GFDPIDHTKVDERLGSWDDVAELSKT.HNIMVDAIVNHMSWESKQFQDVLAKGEESEYYP
TtSPP    51   GFAPITYSEIEPKFGTWYDIKKMAEN.FDILLDLMVNHVSRRSIYFQDFLKKGRKSEYAD
IcSPP    60   GFSIIDYDLN.SDLASAEDLAALDELGIMLKFDMVLNHLSVGSPQFQDLLKHGDDSAFRD

BaSP    111   MFLTMSSVFPNGATEE..........DLAGIYRPRPGLPFTHYKF..AGKTRLVWVSFTP
TtSPP   110   MFITLDKLWKDGKPVK..........GDIEKMFLRRTLPYSTFKIEETGEEEKVWTTFGK
IcSPP   119   FFIDWNEFWEGEGELHADGHVVPSPEHLDRLFMRKPGLPILQVRFP.DGSDRFYWNTFYQ

BaSP    159   ............QQVDIDTDSDKGWEYLMSIFDQMAASHVSYIRLDAVGYGAKEAGTSCF
TtSPP   160   T........DPSEQIDLDVNSHLVREFLLEVFKTFSNFGVKIVRLDAVGYVIKKIGTSCF
IcSPP   178   RVETIDGERSYLGQMDLNAESPRVWTFYRETFEKLARYGAKIVRLDAFAYLHKAVGDTNF

BaSP    207   M.TPKTFKLISRLREEGVKRGLEILIEVHSYYKKQ..VEIASKVDRVYDFALPPLLLHAL
TtSPP   212   FVEPEIYEFLDWAKGQAASYGIELLLEVHSQFEVQ..YKLAERGFLIYDFILPFTVLYTL
IcSPP   238   FNTPGTWDHLDRLRTISEENGLVLLPEIHGEYGTKIHEELSDRDYPVYDFFFPGLVIDAI

BaSP    264   STGHVEPVAHWTDI...RPNNAVTVLDTHDGIGVIDIGSDQLDRSLKGLVPDEDVDNLVN
TtSPP   270   INKSNEMLYHYLKN...RPINQFTMLDCHDGIPVKPD........LDGLIDTKKAKEVVD
IcSPP   298   DSASNTHLLRWIDEIIERDIATVNMLGCHDGIPVIDLKGGPT...GQGLLPDATIEAMIS

BaSP    321   TIHANTHG..ESQAATGAAASNLDLYQVNSTYYSALGCNDQHYIAARAVQFFLPGVPQVY
TtSPP   319   ICVQRGANLSLIYGDKYKSEDGFDVHQINCTYYSALNCDDDAYLAARAIQFFTPGIPQVY
IcSPP   355   RLLERGGRVKNLYGADG...TKVSYYQVNATFFSALGESDARLRLARAIQLFVPGTPQVW
                                        Loop A

BaSP    379   YVGALAGKNDMELLRKT..NNGRDINRHYYSTAEIDENLKRPVVKALNALAKFRNELDAF
TtSPP   379   YVGLLAGVNDFEAVKKT..KEGREINRHNYGLKEIEESVQKNVVQRLLKLIRFRNEYEAF
IcSPP   412   YLDLFAGANDVEAADRAGADGHKEINRTNLSAADVEAGLARPIVLDQLEMIRLRNASPAF

BaSP    437   DGTFSYTTDDDTSISFTWRGETSQATLTFEPKRGLGVDNTTPVAMLEWEDSAGDHRSDDL
TtSPP   437   NGEFFIEDCRKDEIRLTWKKDDKRCSLFIDLKTYK........TTIDYINENGEEVKY.L
IcSPP   472   DGRFEVVPTDDTRLQLRWQNGSTVALLDADLATERFT.......ITHEHDGHTEILGYD.

BaSP    497   IANPPVVA
TtSPP   488   V.......
IcSPP         ........
```

Figure S4 Multiple sequence alignment for BaSP, TtSPP and IcSPP. A position is framed in blue if the majority of its residues are similar. Identical residues are shown in bold, similar residues are shown in red. Loop A is indicated with a grey bar below the sequence. The alignment was visualised using ESPript 3.0 (http://espript.ibcp.fr).

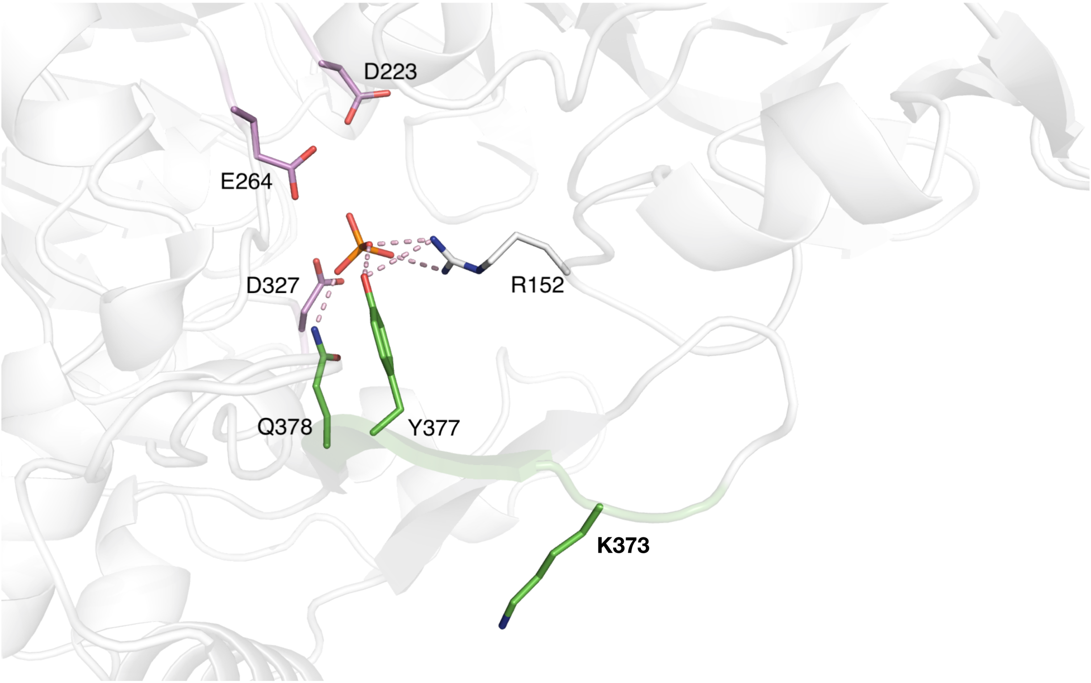

Figure S5 Position of the conserved residue Lys373 in the crystal structure of IcSPP. Shown for reference: catalytic residues (purple), bound phosphate ion and the residues binding the phosphate ion. Loop A is colored in green.

Table S1 Compounds evaluated as substrates for IcSPP.

Reaction	Substrate
Phosphorolysis	Sucrose
	Sucrose 6^F-phosphate
	2-*O*-Glucosylglycerol
	2-*O*-Glucosylglycerate
Synthesis	D-Glucose
	D-Mannose
	D-Galactose
	D-Fructose
	D-Tagatose
	D-Xylose
	D-Ribose
	D-Arabinose
	D-Psicose
	L-Sorbose
	L-Fucose
	L-Arabinose
	L-Rhamnose
	L-Xylose
	L-Ribose
	Gluconic acid
	Glucuronic acid
	Galacturonic acid
	Glucosamine
	Galactosamine
	N-Acetyl-D-Glucosamine
	N-Acetyl-D-Galactosamine
	D-Glyceric Acid
	Glycerol
	D-Mannitol
	D-Sorbitol
	D-Xylitol
	D-Ribitol
	Erythritol
	Trehalose
	Kojibiose
	Maltose
	Sophorose
	Cellobiose
	Sucrose

Table S2 Data collection and refinement statistics.

	IcSPP	TtSPP
**Data collection**		
PDB code	6S9U	6S9V
Beamline	ESRF ID30A-3	ESRF ID30A-3
Wavelength (Å)	0.9677	0.9677
Space group	$C222_1$	$P2_12_12_1$
Unit cell (Å)	63.4, 92.1, 180.0	79.3, 83.7, 147.4
Resolution (Å)	46 – 2.05 (2.11 – 2.05)	38 – 1.83 (1.86 – 1.83)
Total reflections	169930	461327
Unique reflections	33373	86925
Completeness (%)	99.6 (100.0)	99.8 (99.8)
Redundancy	5.1 (5.1)	5.3 (5.5)
Mean $I/\sigma(I)$	9.7 (2.1)	12.7 (2.3)
R_{merge}	0.096 (0.749)	0.068 (0.605)
$CC_{1/2}$	0.995 (0.638)	0.998 (0.801)
**Refinement**		
non-H atoms AU		
Protein, solvent, other	4141, 313, 51	8075, 464, 117
R_{work}, R_{free}	0.169, 0.202	0.158, 0.184
r.m.s.d. from ideal values		
Bond lengths (Å)	0.005	0.005
Bond angles (°)	0.7	0.8
B-factors (Å^2)		
Protein, solvent, other	30.0, 32.6, 48.8	30.8. 33.9, 58.8
Ramachandran plot (%)		
Favored, allowed, outliers	97.1, 2.7, 0.2	97.3, 2.7, 0.0
Ramachandran plot (%)		
Favored, allowed, outliers	97.1, 2.7, 0.2	97.3, 2.7, 0.0

Highest resolution shell in parenthesis. AU is asymmetric unit.

Table S3 Protein sequences for the sucrose 6'-phosphate phosphorylases from *Ilumatobacter coccineus* (IcSPP) and from *Thermoanaerobacterium thermosaccharolyticum* (TtSPP). The His_6-tag and linker sequences are underlined.

Enzyme	Sequence
IcSPP	MTHAGMKASLPNRVMLNAYPDSIDGDLAGTVRMLQRPEFTDAFGLFY<br>VLPSIFNSDLDRGFSIIDYDLNSDLASAEDLAALDELGIMLKFDMVL<br>NHLSVGSPQFQDLLKHGDDSAFRDFFIDWNEFWEGEGELHADGHVVP<br>SPEHLDRLFMRKPGLPILQVRFPDGSDRFYWNTFYQRVETIDGERSY<br>LGQMDLNAESPRVWTFYRETFEKLARYGAKIVRLDAFAYLHKAVGDT<br>NFFNTPGTWDHLDRLRTISEENGLVLLPEIHGEYGTKIHEELSDRDY<br>PVYDFFFPGLVIDAIDSASNTHLLRWIDEIIERDIATVNMLGCHDGI<br>PVIDLKGGPTGQGLLPDATIEAMISRLLERGGRVKNLYGADGTKVSY<br>YQVNATFFSALGESDARLRLARAIQLFVPGTPQVWYLDLFAGANDVE<br>AADRAGADGHKEINRTNLSAADVEAGLARPIVLDQLEMIRLRNASPA<br>FDGRFEVVPTDDTRLQLRWQNGSTVALLDADLATERFTITHEHDGHT<br>EILGYDLEHHHHHH
TtSPP	MGGSHHHHHHGMASMALKNKVQLITYPDSLGGNLKTLNDVLEKYFSD<br>VFGGVHILPPFPSSGDRGFAPITYSEIEPKFGTWYDIKKMAENFDIL<br>LDLMVNHVSRRSIYFQDFLKKGRKSEYADMFITLDKLWKDGKPVKGD<br>IEKMFLRRTLPYSTFKIEETGEEEKVWTTFGKTDPSEQIDLDVNSHL<br>VREFLLEVFKTFSNFGVKIVRLDAVGYVIKKIGTSCFFVEPEIYEFL<br>DWAKGQAASYGIELLLEVHSQFEVQYKLAERGFLIYDFILPFTVLYT<br>LINKSNEMLYHYLKNRPINQFTMLDCHDGIPVKPDLDGLIDTKKAKE<br>VVDICVQRGANLSLIYGDKYKSEDGFDVHQINCTYYSALNCDDDAYL<br>AARAIQFFTPGIPQVYYVGLLAGVNDFEAVKKTKEGREINRHNYGLK<br>EIEESVQKNVVQRLLKLIRFRNEYEAFNGEFFIEDCRKDEIRLTWKK<br>DDKRCSLFIDLKTYKTTIDYINENGEEVKYLV
